# Infants Receiving a Single Dose of Nirsevimab to Prevent RSV Do Not Have Evidence of Enhanced Disease in Their Second RSV Season

**DOI:** 10.1093/jpids/piad113

**Published:** 2024-01-14

**Authors:** Ron Dagan, Laura L Hammitt, Beatriz Seoane Nuñez, Manuel Baca Cots, Miroslava Bosheva, Shabir A Madhi, William J Muller, Heather J Zar, Yue Chang, Alexander Currie, Amy Grenham, Manish Shroff, Therese Takas, Vaishali S Mankad, Amanda Leach, Tonya Villafana

**Affiliations:** The Shraga Segal Department of Microbiology, Immunology and Genetics, Faculty of Health Sciences at the Ben-Gurion University of the Negev, Beer-Sheva, Israel; Department of International Health, Johns Hopkins University, Baltimore, Maryland, USA; Biometrics, Vaccines & Immune Therapies, BioPharmaceuticals R&D, AstraZeneca, Madrid, Spain; Paediatric Service, Quirónsalud Málaga Hospital, Málaga, Spain; Paediatrics, University Multiprofile, Hospital for Active Treatment, St. George Medical University, Plovdiv, Bulgaria; South African Medical Research Council Vaccines and Infectious Diseases Analytics Research Unit and African Leadership in Vaccinology Expertise, Faculty of Health Sciences, University of the Witwatersrand, Johannesburg, South Africa; Infectious Diseases, Ann and Robert H. Lurie Children’s Hospital of Chicago, Chicago, Illinois, USA; Stanley Manne Children’s Research Institute, Northwestern University Feinberg School of Medicine, Chicago, Illinois, USA; Department of Paediatrics and Child Health, Red Cross Children’s Hospital, and the Medical Research Council Unit on Child and Adolescent Health, University of Cape Town, Cape Town, South Africa; Clinical Development, Vaccines & Immune Therapies, BioPharmaceuticals R&D, AstraZeneca, Gaithersburg, Maryland, USA; Clinical Development, Vaccines & Immune Therapies, BioPharmaceuticals R&D, AstraZeneca, Cambridge, UK; Clinical Development, Vaccines & Immune Therapies, BioPharmaceuticals R&D, AstraZeneca, Gaithersburg, Maryland, USA; Patient Safety, Chief Medical Office, R&D, AstraZeneca, Waltham, Massachusetts, USA; Clinical Development, Vaccines & Immune Therapies, BioPharmaceuticals R&D, AstraZeneca, Gaithersburg, Maryland, USA; Clinical Development, Vaccines & Immune Therapies, BioPharmaceuticals R&D, AstraZeneca, Durham, North Carolina, USA; Clinical Development, Vaccines & Immune Therapies, BioPharmaceuticals R&D, AstraZeneca, Gaithersburg, Maryland, USA; Clinical Development, Vaccines & Immune Therapies, BioPharmaceuticals R&D, AstraZeneca, Gaithersburg, Maryland, USA

**Keywords:** immunization, monoclonal antibody, nirsevimab, respiratory syncytial virus

## Abstract

To characterize nirsevimab in the prevention of RSV, children from the Phase 3 MELODY trial were followed through their second RSV season. No increase in medically attended RSV lower respiratory tract infections or evidence of antibody-dependent enhancement of infection or disease severity was found for nirsevimab vs placebo recipients.

Clinical Trial Registration: Clinicaltrials.gov, NCT03979313, https://clinicaltrials.gov/ct2/show/NCT03979313.

## Teaser

No increase in the incidence of medically attended RSV lower respiratory tract infection or evidence of antibody-dependent enhancement of disease was found among nirsevimab recipients during their second RSV season in the Phase 3 MELODY trial compared with placebo recipients.

Respiratory syncytial virus (RSV) is the leading cause of lower respiratory tract infection (LRTI) among infants worldwide, and a leading cause of hospital admission in the first year of life [1, 2]. Three approaches to the prevention of RSV have been pursued: passive immunization of infants through the direct administration of antibodies; maternal vaccination during pregnancy with transplacental transfer; or active immunization of infants [3]. However, binding of non-neutralizing antibodies or antibodies binding to viral antigens at sub-neutralizing concentrations without adequately blocking or clearing infection has the potential to lead to the promotion of antibody-dependent enhancement of infection (ADE of infection) or enhanced disease severity (ADE of disease) [4, 5]. In the 1960s, enhanced disease severity was observed following administration of a formalin-inactivated RSV vaccine. Infants who were seronegative to RSV before vaccination experienced RSV LRTI more frequently and with more severe disease upon subsequent natural RSV infection, with 80% of vaccine recipients requiring hospitalization (vs 5% of the control group) and 2 fatalities among vaccinated infants [6]. While the risk is greatest following active immunization, theoretically, any of these approaches to RSV prevention could result in ADE. To date, ADE has not been observed with the monoclonal antibody palivizumab, initially approved in 1998 for the prevention of serious lower respiratory tract RSV disease in infants at higher risk for severe RSV disease [7, 8]. Nirsevimab, an extended half-life recombinant human IgG1 kappa monoclonal antibody, binds the RSV prefusion (pre-F) protein at the highly conserved antigen site Ø, locking it in the pre-F conformation to block viral entry into host cells [9, 10]. The extended half-life was engineered by substituting 3 amino acids (M252Y/S254T/T256E; YTE) within the Fc region of nirsevimab to give a >3-fold longer serum half-life than a typical monoclonal antibody of 11–30 days [11] to approximately 70 days in healthy late preterm and term infants [9, 12]. Nonclinical studies evaluating the potential for ADE in a cotton rat model of RSV using nirsevimab without YTE found no evidence of ADE at any dose evaluated, including doses below the amount needed to protect animals against RSV challenge [9]. In the full-enrollment cohort of the Phase 3, randomized, double-blind, placebo-controlled MELODY trial (*n* = 3012; NCT03979313), nirsevimab demonstrated an efficacy of 76.4% (95% confidence interval 62.3–85.2) against medically attended (MA) RSV LRTI over 151 days, with a favorable safety profile to Day 361 [13]. Children from the MELODY full-enrollment cohort have now been followed through their second RSV season (remaining blinded) to evaluate the theoretical risk of ADE and whether prophylaxis with nirsevimab results in a shift of the burden of disease to the second year of life.

## METHODS

Details of the MELODY study design and methodology, including case definitions with increasing severity of MA RSV LRTI, have been reported previously [12, 13]. Briefly, MELODY enrolled healthy infants born at ≥35 weeks 0 days gestational age. Participants were randomized 2:1 to a single intramuscular dose of nirsevimab (50 mg for infants <5 kg; 100 mg for infants ≥5 kg) or placebo, administered at the start of their first RSV season; no dose was given prior to their second season. All children were under continuous passive surveillance for MA RSV LRTI from study start through Day 511, with the first season being Day 1 to Day 151 post-dose and the second season being Day 362 to Day 511 post-dose. Methodology was consistent over the study period: all children who presented with clinical evidence of LRTI or required hospitalization for any respiratory tract infection (including upper and LRTI) were assessed, with clinical symptoms and signs documented, oxygen saturation measured, and specimens taken for RSV testing at a reference laboratory. Given the atypical seasonal pattern of RSV circulation due to the coronavirus disease 2019 (COVID-19) pandemic during the study period, we report all cases, including those that occurred in the interval between seasons. According to protocol, RSV infection was confirmed by central testing (reverse transcription polymerase chain reaction assay; Lyra RSV plus hMPV, Quidel); results of local clinical testing were recorded when available. Case definitions for events due to RSV comprised MA RSV LRTI, MA RSV LRTI with hospitalization, very severe MA RSV LRTI, MA RSV-associated LRTI on any test result, and hospitalization for any respiratory illness due to RSV on any test result; events of any cause (inclusive of RSV) comprised MA LRTI of any cause and hospitalization for any respiratory illness of any cause. The protocol was approved by the institutional ethics review board or ethics committee at each participating site, and all participants provided written informed consent.

## RESULTS

Overall, 3012 infants were randomized between July 23, 2019, and October 22, 2021 across 211 sites in 31 countries in both the Northern and Southern hemispheres (intent-to-treat population; enrollment was paused during the peak of the COVID-19 pandemic between March 15, 2020 [the end of the first season of observation in the Northern hemisphere], and April 9, 2021). Of these, 2911 (97%; nirsevimab: *n* = 1944 [97%]; placebo: *n* = 967 [96%]) completed to Day 361 and were followed up through their second RSV season (Supplementary Figure 1) without redosing; 2796 (93%) infants randomized completed to Day 511 (nirsevimab: *n* = 1873 [93%]; placebo: *n* = 923 [92%]).

During their second RSV season, 134 (6.9%) nirsevimab and 71 (7.3%) placebo recipients had an MA LRTI of any cause (including RSV), while 21 (1.1%) nirsevimab and 11 (1.1%) placebo recipients were hospitalized for any respiratory illness of any cause (Table 1). Using a more sensitive case definition encompassing all events of RSV disease, events during the infants’ second season were balanced by treatment group, occurring in 35 (1.8%) nirsevimab recipients and 20 (2.1%) placebo recipients, of which 10 (0.5%) and 6 (0.6%) cases required hospitalization in each group, respectively (Supplementary Table 1). Among the LRTIs confirmed as RSV positive, 19 (1.0%) nirsevimab and 10 (1.0%) placebo recipients had an MA RSV LRTI meeting the per-protocol case definition, with 3 recipients in each group requiring hospitalization, all of which were classified as very severe MA RSV LRTI (0.2% and 0.3%, respectively; Table 1).

**Table 1.** Incidence of RSV-Associated Respiratory Disease in the First and Second RSV Seasons (ITT Population^a^)

	First RSV Season (Through 151 Days Post-dose)		Second RSV Season^b^ (362–511 Days Post-dose)	
Disease Event (*n* [%])	Nirsevimab (*N* = 2009)	Placebo (*N* = 1003)	Nirsevimab (*N* = 1944)	Placebo (*N* = 967)
Events due to RSV				
Medically attended RSV LRTI^c^	24 (1.2)	54 (5.4)	19 (1.0)	10 (1.0)
Medically attended RSV LRTI with hospitalization^c^	9 (0.4)	20 (2.0)	3 (0.2)	3 (0.3)
Medically attended RSV LRTI (very severe)^d^	7 (0.3)	17 (1.7)	3 (0.2)	3 (0.3)
Medically attended RSV-associated LRTI on any test result^e^^,^^f^	34 (1.7)	75 (7.5)	35 (1.8)	20 (2.1)
Hospitalization for any respiratory illness due to RSV on any test result^f^^,^^g^	15 (0.7)	26 (2.6)	10 (0.5)	6 (0.6)
Events of any cause (inclusive of RSV)				
Medically attended LRTI of any cause^e^	172 (8.6)	139 (13.9)	134 (6.9)	71 (7.3)
Hospitalization for any respiratory illness of any cause^g^	45 (2.2)	37 (3.7)	21 (1.1)	11 (1.1)

Abbreviations: ITT, intent-to-treat; IV, intravenous; LRTI, lower respiratory tract infection; RSV, respiratory syncytial virus.

^a^ITT population included all participants who underwent randomization.

^b^In the second season, the number of ITT participants who were followed up for ≥362 days post-dose was used as the denominator for calculation of incidence.

^c^Per-protocol definition of medically attended RSV LRTI.

^d^Restricted to those children requiring oxygen supplementation or IV fluids for management of medically attended RSV LRTI (per protocol definition).

^e^Medically attended LRTI in investigators judgment, regardless of whether they met all the criteria for the per-protocol case definition of a medically attended LRTI.

^f^Any test result refers to either the central reference test for the trial or a local test performed in the context of clinical care.

^g^Any respiratory illness includes both upper respiratory tract infection and LRTI.

To ensure a comprehensive assessment of RSV-associated respiratory disease and provide the opportunity to detect ADE during a time when levels of nirsevimab would be expected to be waning, the incidence of RSV-associated respiratory disease between Seasons 1 and 2 (152–361 days post-dose) was also evaluated (Supplementary Table 2); the incidence of events was near equivalent for nirsevimab and placebo across case definitions of disease.

## DISCUSSION

In this study of children in their second RSV season who received nirsevimab prior to their first RSV season, the incidence of RSV LRTI was low and there was no increase in the severity of disease relative to placebo. Thus, in the predicted conditions of sub-neutralizing concentrations of nirsevimab, there was no indication of ADE in this population. Furthermore, the similar incidence of RSV LRTI among nirsevimab and placebo recipients in their second RSV season suggests that prophylaxis with nirsevimab to protect against RSV disease in the first season does not result in a shift of the burden of disease to the second year of life. This is in keeping with evidence that nirsevimab does not inhibit an immune response to natural RSV infection [14].

RSV circulates seasonally for approximately 150 days, or 5 months of the year in temperate regions, consequently, the dates of the second season varied depending on whether the child lived in the Northern or Southern hemisphere and when they were enrolled. However, regardless of location, a limitation of these data is that RSV circulation in 2020–2021 was lower than usual due to the wide implementation of nonpharmacologic interventions in response to the COVID-19 pandemic [15]. Furthermore, there is a lower risk for RSV LRTI in the second year of life. Consequently, to capture as many potential cases as possible and to enable the most comprehensive and robust analysis, we have provided data from any test across both seasons and between seasons (152–361 days post-dose), including all-cause data and data from the most sensitive case definitions.

In conclusion, these data do not indicate an increase in the incidence or severity of events of RSV LRTI in the second year of life among infants administered nirsevimab prior to their first RSV season. Although ADE is a theoretical concern of RSV prevention approaches, there was no evidence of such an effect in infants who received nirsevimab.

## Supplementary Material

piad113_suppl_Supplementary_Figure_S1_Tables_S1-S2

## Data Availability

Data underlying the findings described in this manuscript may be obtained in accordance with AstraZeneca’s data sharing policy described at https://astrazenecagrouptrials.pharmacm.com/ST/Submission/Disclosure. Data for studies directly listed on Vivli can be requested through Vivli at www.vivli.org. Data for studies not listed on Vivli could be requested through Vivli at https://vivli.org/members/enquiries-about-studies-not-listed-on-the-vivli-platform/. AstraZeneca Vivli member page is also available outlining further details: https://vivli.org/ourmember/astrazeneca/.

## Notes

***Financial support*:** This study was supported by AstraZeneca and Sanofi. AstraZeneca was involved in the trial design; the collection, analysis, and interpretation of the data; and the writing of the manuscript. Nirsevimab is being developed and commercialized in partnership between AstraZeneca and Sanofi.

***Potential conflicts of interest*:** R.D. has received grants or contracts from AstraZeneca, Merck, and Pfizer; consulting fees from Merck and Pfizer; and honoraria from GSK, Merck, Pfizer, and Sanofi Pasteur. L.L.H has received grants to her institution from AstraZeneca, Merck, and Pfizer. M.B.C. and M.B. report no conflicts of interest. S.A.M. has received grants or contracts from the Bill and Melinda Gates Foundation, GSK, MinervaX, Pfizer, and the South African Medical Research Council; has received payments or honoraria from the Bill and Melinda Gates Foundation; and has participated in data safety monitoring boards or advisory boards for PATH and CAPRISA. W.J.M. has received grants or contracts from Ansun, Astellas, AstraZeneca, Eli Lilly, Enanta Pharmaceuticals, Genentech, Gilead, Janssen, Karius, Melinta, Merck, Moderna, Nabriva, Paratek, Pfizer, and Tetraphase; consulting fees from Finley Law Firm and Seqirus; has received payment or honoraria from Contemporary Pediatrics; and has participated in data safety monitoring boards or advisory boards for Adagio Therapeutics, AstraZeneca, ProventionBio, and Sanofi Pasteur. H.J.Z. has received grants or contracts from AstraZeneca, MSD, and Pfizer; and has received payment or honoraria from Merck. B.S.N., Y.C., A.C, A.G., M.S., T.T., V.S.M., and T.V. are employees of AstraZeneca and may hold stock or stock options. A.L. is a former employee of AstraZeneca and may hold stock or stock options.
