# Supplementary material for: Infants Receiving a Single Dose of Nirsevimab to Prevent RSV Do Not Have Evidence of Enhanced Disease in Their Second RSV Season
Source: J Pediatric Infect Dis Soc. 2024 Jan 14;13(2):144–7. doi: 10.1093/jpids/piad113 (PMC10896255; doi:10.1093/jpids/piad113)
Supplement: piad113_suppl_Supplementary_Figure_S1_Tables_S1-S2 [file piad113_suppl_supplementary_figure_s1_tables_s1-s2.docx]

# **SUPPLEMENTARY MATERIAL**

**Supplementary Table 1. Participants With Hospitalization for Any Respiratory Illness Due to RSV on Any Test Result During their Second RSV Season (362–511 Days Post-dose; ITT Population^a^)**

| Treatment | Age at Randomization/Onset (Months) | Sex | Race | Country | Days  to Event^b^ | Number of Days Hospitalized | Oxygen Supplementation | Other Respiratory Support | Dehydration Due to Respiratory Distress |
| --- | --- | --- | --- | --- | --- | --- | --- | --- | --- |
| Placebo | 5.9/18.4 | Female | M | USA | 381 | 3 | No | No | Yes^c^ |
|  | 1.1/14.5 | Male | W | Spain | 409 | 7 | Yes | No | Yes^c^ |
|  | 2.3/14.3 | Female | W | Spain | 366 | 6 | Yes | Yes^d^ | No |
|  | 0.7/16.6 | Female^e^ | W | Belgium | 484 | 12 | Yes | No | No |
|  | 1.6/17.8 | Female | O | Panama | 495^f^ | 7 | Yes | No | No |
|  | 5.0/20.3 | Female | N | Panama | 467^g^ | 12 | Yes | No | No |
| Nirsevimab | 6.1/19.7 | Male | W | Argentina | 415 | 6 | Yes | No | No |
|  | 5.9/18.4 | Male | B | USA | 381 | 3 | No | No | Yes^c^ |
|  | 5.1/18.9 | Female | W | Australia | 422 | 2 | Yes | No | No |
|  | 0.7/16.6 | Female^e^ | W | Belgium | 484 | 17 | Yes | No | No |
|  | 0.03/15.2 | Male | W | Israel | 463 | 3 | No | No | No |
|  | 4.8/20.4 | Male | N | USA | 477 | 2 | Yes | No | No |
|  | 5.5/21.2 | Male | O | Panama | 480 | 5 | Yes | No | No |
|  | 1.6/16.3 | Male | A | Japan | 447 | 5 | Yes | No | Yes^c^ |
|  | 2.7/15.6 | Female | W | Spain | 392 | 5 | Yes | No | No |
|  | 5.2/18.8 | Female | M | USA | 413 | 2 | Yes | No | No |

Abbreviations: A, Asian, B, Black; ITT, intent-to-treat; LRTI, lower respiratory tract infection; M, multiple; MA, medically attended; N, American Indian or Alaska Native; O, Other; RSV, respiratory syncytial virus; W, White.

^a^Included all participants who underwent randomization and were followed through to their second season.

^b^Days between dosing and being seen by the healthcare provider for the event.

^c^Received treatment with IV fluids.

^d^Continuous positive airway pressure.

^e^Participants are twins.

^f^One participant also had an MA RSV LRTI that did not meet the per-protocol case definition in their 1st RSV season (26 days post-dose) .

^g^One participant also had an event meeting the per-protocol case definition of MA RSV LRTI in their 1st RSV season (16 days post-dose).

**Supplementary Table 2. Incidence of RSV-Associated Respiratory Disease Between the First and Second RSV Seasons (ITT Population^a^)**

|  | Between 1st and 2nd Season  152–361 Days Post-dose | |
| --- | --- | --- |
| **Disease Event (n [%])** | Nirsevimab  (N = 1977) | Placebo  (N = 985) |
| Events due to RSV |  | |
| Medically attended RSV LRTI^b^ | 16 (0.8) | 13 (1.3) |
| Medically attended RSV LRTI with hospitalization^b^ | 2 (0.1) | 2 (0.2) |
| Medically attended RSV LRTI (very severe)^c^ | 2 (0.1) | 1 (0.1) |
| Medically attended RSV-associated LRTI on any test result^d,e^ | 25 (1.3) | 19 (1.9) |
| Hospitalization for any respiratory illness due to RSV on any test result^e,f^ | 3 (0.2) | 2 (0.2) |
| Events of any cause (inclusive of RSV) |  | |
| Medically attended LRTI of any cause^d^ | 143 (7.2) | 67 (6.8) |
| Hospitalization for any respiratory illness of any cause^f^ | 37 (1.9) | 10 (1.0) |

Abbreviations: ITT, intent-to-treat; IV, intravenous; LRTI, lower respiratory tract infection; RSV, respiratory syncytial virus.

^a^ITT population (all participants who underwent randomization and were followed up for ≥151 days post-dose) was used as the denominator for calculation of incidence.

^b^Per-protocol definition of medically attended RSV LRTI.

^c^Restricted to those children requiring oxygen supplementation or IV fluids for management of medically attended RSV LRTI (per-protocol definition).

^d^Medically attended LRTI in investigators judgment, regardless of whether they met all the criteria for the per-protocol case definition of a medically attended LRTI.

^e^Any test result refers to either the central reference test for the trial or a local test performed in the context of clinical care.

^f^Any respiratory illness includes both upper respiratory tract infection and LRTI.


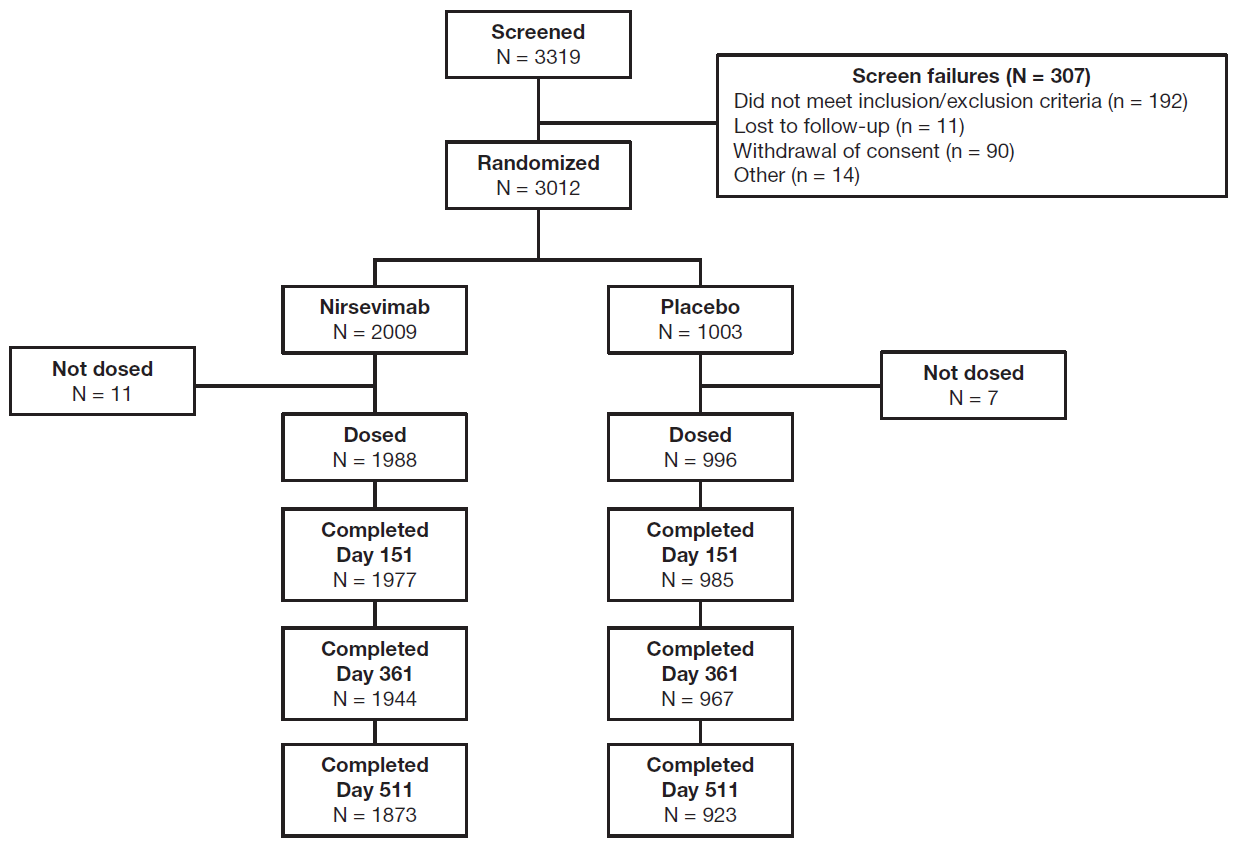


**Supplementary Figure 1.** CONSORT diagram. Discontinuations during the course of the trial included, lost to follow-up (nirsevimab, n = 61 [3.0%]; placebo, n = 28 [2.8%]), withdrawn by parent/legal representative (nirsevimab, n = 44 [2.2%]; placebo, n = 35 [3.5%]), COVID-19 pandemic (nirsevimab, n = 3 [0.1%]; placebo, n = 1 [0.1%]), death (nirsevimab, n = 5 [0.2%]; placebo, n = 0 [0%]), and other (nirsevimab, n = 23 [1.1%]; placebo, n = 16 [1.6%]).

Abbreviation: COVID-19, coronavirus disease 2019.
